# Use of mental health services among disaster survivors: predisposing factors

**DOI:** 10.1186/1471-2458-7-173

**Published:** 2007-07-24

**Authors:** Dirk-Jan den Ouden, Peter G van der Velden, Linda Grievink, Mattijn Morren, Anja JE Dirkzwager, C Joris Yzermans

**Affiliations:** 1 Netherlands Institute for Health Services Research (NIVEL), Utrecht, The Netherlands; 2 Institute for Psychotrauma (IVP), Zaltbommel, The Netherlands; 3 Dutch National Institute for Public Health and the Environment (RIVM), Utrecht, The Netherlands

## Abstract

**Background:**

Given the high prevalence of mental health problems after disasters it is important to study health services utilization. This study examines predictors for mental health services (MHS) utilization among survivors of a man-made disaster in the Netherlands (May 2000).

**Methods:**

Electronic records of survivors (n = 339; over 18 years and older) registered in a mental health service (MHS) were linked with general practice based electronic medical records (EMRs) of survivors and data obtained in surveys. EMR data were available from 16 months pre-disaster until 3 years post-disaster. Symptoms and diagnoses in the EMRs were coded according to the International Classification of Primary Care (ICPC). Surveys were carried out 2–3 weeks and 18 months post-disaster, and included validated questionnaires on psychological distress, post-traumatic stress reactions and social functioning. Demographic and disaster-related variables were available. Predisposing factors for MHS utilization 0–18 months and 18–36 months post-disaster were examined using multiple logistic regression models.

**Results:**

In multiple logistic models, adjusting for demographic and disaster related variables, MHS utilization was predicted by demographic variables (young age, immigrant, public health insurance, unemployment), disaster-related exposure (relocation and injuries), self-reported psychological problems and pre- and post-disaster physician diagnosed health problems (chronic diseases, musculoskeletal problems). After controlling for all health variables, disaster intrusions and avoidance reactions (OR:2.86; CI:1.48–5.53), hostility (OR:2.04; CI:1.28–3.25), pre-disaster chronic diseases (OR:1.82; CI:1.25–2.65), injuries as a result of the disaster (OR:1.80;CI:1.13–2.86), social functioning problems (OR:1.61;CI:1.05–2.44) and younger age (OR:0.98;CI:0.96–0.99) predicted MHS utilization within 18 months post-disaster. Furthermore, disaster intrusions and avoidance reactions (OR:2.29;CI:1.04–5.07) and hostility (OR:3.77;CI:1.51–9.40) predicted MHS utilization following 18 months post-disaster.

**Conclusion:**

This study showed that several demographic and disaster-related variables and self-reported and physician diagnosed health problems predicted post-disaster MHS-use. The most important factors to predict post-disaster MHS utilization were disaster intrusions and avoidance reactions and symptoms of hostility (which can be identified as symptoms of PTSD) and pre-disaster chronic diseases.

## Background

Most disaster survivors experience a number of responses in the aftermath of a disaster, such as feelings of sadness, anger, guilt, numbness and sleep disturbances. These responses can be seen as normal stress reactions to an abnormal situation. However, some survivors are more affected than others and develop serious mental health problems, such as anxiety disorders, depression and post-traumatic stress disorder (PTSD) [1-4]. PTSD is the most common psychiatric disorder after a traumatic event and is characterised by having three categories of symptoms: intrusion, avoidance and hyperarousal. Intrusions are manifested in a preoccupation with the disaster, repeated thoughts about the event, vivid memories accompanied by painful emotions or nightmares. Avoidance reactions such as emotional numbness, refusal to talk about it and avoidance of locations reminding of the traumatic event are considered as attempts to block out the intrusions. Hyperarousal is characterised by a state of nervousness, accelerated heart beat, difficulty sleeping.

Treatment for mental disorders is important to reduce symptoms and to prevent future problems. An important impulse to prevent and conquer disaster health problems is the delivery of specific services to deal with the needs of the affected population. Disaster mental health services (MHS) are aimed at returning community equilibrium by restoring psychological and social functioning of individuals and limiting the occurrence and severity of these adverse disaster-related health problems [5]. Treatments for different disaster-related disorders have been found effective in reducing symptoms [6-8].

Several studies on MHS utilization following disaster have been carried out in the past. For example, Boscarino concluded that 10% of the Manhattan residents increased their mental health visits within 30 days following the September 11^th ^terrorist attacks compared to the month before the disaster [9].

For effective public health planning, it is essential to determine factors that predispose to MHS utilization. Two recent reviews have focused on predictors or predisposing factors for MHS-use [10,11]. In a critical review of 34 studies regarding health services use among trauma survivors, including disaster survivors, Elhai et al demonstrated that survivors with a previous trauma history and female trauma survivors (veteran studies excluded) more frequently used MHS than their counterparts. They showed that (subclinical) PTSD was clearly related to increased use of MHS. Furthermore, they found various results for different subgroups, such as age group (either unrelated to MHS use or older age predicted MHS use), racial group (either no association or immigrants were less likely to use MHS), unemployment (predicting greater MHS use or no relation) [10]. Gavrilovic found that the most important factors associated with treatment seeking appear to be a higher level of psychopathology, the type and level of the traumatic event and sociodemographic characteristics [11].

However, some of the studies reviewed were based on self-reported data and applied only descriptive/unadjusted statistics. The reviewed studies did not differentiate between different post-disaster periods regarding the factors associated with seeking treatment from MHS.

To our knowledge, few studies have used electronic MHS records as an outcome of MHS utilization in combination with predisposing variables from both electronic medical records (EMRs) *and *self-reported questionnaires. A recent study conducted after hurricane Katrina among evacuated veterans showed the importance of electronic records regarding health care delivery after the disaster [12].

The present study adds to the existing literature as it is based on a population of disaster survivors who were all registered with a GP. Data from both (pre- and post-disaster) electronic records and post-disaster self-reports were available and were tested in multivariate models in order to control for possible confounders. The electronic records of one MHS, which was specially implemented for disaster survivors only, was used as an outcome variable. Furthermore, we analysed two different post-disaster periods in which survivors sought help to examine possible differences in factors associated with help seeking.

The aim of the present study is to examine predisposing factors for MHS use in survivors of a man-made disaster. In addition, we analysed the predisposing factors for MHS-use for two post-disaster periods.

## Methods

### Background

On 13 May 2000, a fireworks depot exploded in the city of Enschede, the Netherlands, which destroyed a large part of the neighbourhood. As a result, 23 people were killed, about 1000 were injured and 1200 lost their homes [13]. Immediately after the disaster, a local community mental health service was implemented exclusively for victims of this disaster. Much attention was given to the availability of this service through public campaigns by leaflets, papers, radio and television to stimulate people with mental problems to seek treatment. Survivors in this MHS received mental health care provided by psychologists, psychiatrists and social workers. After the disaster a large scale study was implemented to explore disaster-related consequences in affected residents involved in the aftermath of the disaster [13]. This study consisted of two different approaches: 1) a longitudinal surveillance using the electronic registration systems of health care providers (i.e. general practitioners, mental health care unit [14,15]. and 2) longitudinal surveys in which affected residents of 18 years and older were invited to participate [16]. In the current study these three different databases (from general practitioners, a mental health service unit and surveys) were combined.

### Design

**Figure 1:**
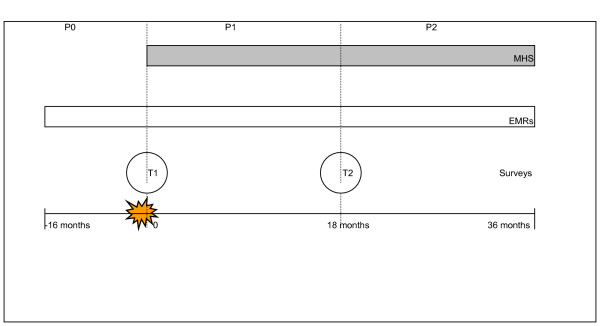
**Study design**. MHS = mental health services; EMRs = electronic medical records; P = period; T = time.

Figure 1 shows the study design. Data from the MHS-electronic records were extracted for the period 13 May 2000 to 13 May 2003. Self-report questionnaires were administered on two occasions. The first measurement (T1) was conducted 2–3 weeks post-disaster, the second measurement (T2) 18 months post-disaster. Participants gave their written informed consent and a Medical Ethical Committee approved the study protocols.

Data from the EMRs of the general practitioners were extracted from 13 January 1999 to 13 May 2003. Data collection procedures were in accordance with the privacy protection guidelines of the Dutch Data Protection Authority.

### Participants

Our target population consisted of all affected residents, 18 years and older, who received help in the specific MHS (n = 1,008) within the study period. For 339 survivors out of this 1008, *both *data from the electronic medical records (EMRs) of their GPs *and *data from the surveys were available. These 339 patients formed the study population (MHS-group). Analyses showed that these 339 patients did not differ from the remaining MHS patients on sex, immigrant status, age, and forced relocation due to damaged housing (an indicator of exposure). 239 out of these 339 survivors who were registered in the MHS unit sought help between 0–18 months post-disaster; 100 persons were registered in the electronic register between 18–36 months post-disaster. The mean duration of the treatment in the MHS was around 7.5 months and survivors had 12 contacts/consultations on average.

Our non-MHS group consisted of 1,197 disaster survivors who were included both in the longitudinal surveillance in general practice (whose EMRs were available) and in the surveys. The non-MHS group did not attend this specific MHS unit.

### Databases and instruments

#### Electronic MHS records

A number of demographic variables (sex, age, immigrant status – defined as first and second generation versus Dutch natives) and information on number of contacts and date of admission was recorded in the electronic database.

#### Electronic medical records

In the Dutch health care system each citizen is registered with one GP who acts as a gatekeeper to secondary care. Information on patients' symptoms and diagnoses was extracted from the electronic medical records (EMRs) of the GP and was registered according to the International Classification of Primary Care (ICPC) [17]. Using individual ICPC codes will result in rather small numbers. Therefore, clusters of ICPC codes were composed according to the type of health problem (eg. psychological problems, chronic diseases, musculoskeletal-, gastrointestinal- and respiratory symptoms) [18]. Prevalence rates were calculated as whether or not a patient consulted the GP in a given period for health problems in a specific cluster.

In addition, information on forced relocation as a result of the disaster and health insurance was available. Until 2006, the Dutch insurance system was divided into public (state run) and private health insurance. Persons were publicly insured when their gross annual income was below a certain level. Therefore, type of health insurance can be used as a proxy for socioeconomic status (SES).

#### Surveys

The following demographic characteristics were used for the present study: marital status and employment status. Furthermore, survivors were asked if they were injured as a result of the disaster and whether they lost a family member/friend or colleague as a result of the disaster.

Participants filled out the following questionnaires on both T1 and T2.

To assess psychological distress the Dutch version of the SCL-90-R was administered which has good psychometric properties [19,20]. Items have a 5-point intensity scale (1 = not at all, 5 = extremely) to assess the severity of several symptoms over the past 7 days. For the purpose of the present study, we used the subscales anxiety (10 items), depression (14 items), hostility (6 items) and somatisation (12 items). The 95^th ^percentile of a Dutch normative sample was used as a cut-off score, indicating a 'very high' score [18]. The internal consistency of the SCL-90-R subscales was satisfactory with Cronbach's alphas ranging from .79 to .95 on both measurements.

To examine disaster intrusions and avoidance reactions, the Dutch version of the Impact of Event Scale (IES) was used, which consists of 15 items that are rated on a 4 point frequency scale (0 = not at all, 5 = often) to assess symptoms over the past 7 days. Reliability and validity of this instrument has been found to be satisfactory [21,22]. A cut-off score of 26 was used for the IES subscales to distinguish low versus high scores [23]. At both measurements the internal consistency was good with Cronbach's alpha coefficients raging from 0.84 to 0.91. The IES 2-factor structure has convergent validity with diagnosed PTSD [21].

To assess social functioning problems as a result of health problems, a subscale of the RAND-36 health survey was used [24]. The subscale consists of 2 items that are rated on a 5 point frequency scale (1 = not al all, 5 = very often) and assess social functioning in the past 2 weeks for T1 and in the past 4 weeks for T2. The scores were dichotomised; a score of 1 represented a score of more than one standard deviation below the average score of a Dutch national sample [25]. Cronbach's alpha coefficients for this sample ranged from .77 to .86 at both measurements.

### Statistical analyses

Group differences on demographic characteristics between MHS users and the non-MHS group were examined using Chi square tests (categorical variables) and t-tests (continuous variables).

To examine which factors predicted post-disaster MHS, we used a multiple logistic regression strategy. In the first model, we examined whether demographic and disaster-related variables predicted MHS use. The following independent variables were entered in the regression analyses simultaneously: sex, age, insurance type, immigrant status, marital status, employment status, forced relocation, injuries as a result of the disaster and whether the lost a family member/friend of colleague as a result of the disaster. Because of low cell frequencies, the latter two variables were combined into one variable for the analysis ('injured'). The adjusted OR and 95% confidence intervals (CI) were reported. In the second model, we examined whether different health measures predicted MHS-use, after controlling for the demographic and disaster-related variables which were entered in the first model. The health-related variables were added separately into the regression model in order to study the adjusted OR (and 95% CI) for that specific variable. ORs adjusted for demographic characteristics and their 95% CI were reported.

To examine significant independent predictors for post-disaster MHS use, a multiple regression analysis was performed in the third (saturated) model in which all variables (demographic-, disaster-related and health-related variables) were entered simultaneously. Multicolinearity was not a factor in the analysis. Backward stepwise logistic regression analysis revealed no differences in significant outcomes compared to results of the fully saturated model. All statistical analyses were carried out using SPSS version 11.5 [26].

## Results

### Sample characteristics

The sample characteristics of the survivors registered with the MHS are presented in table 1. Compared to the non-MHS group, survivors who were registered with the MHS were younger, more often relocated and publicly insured and were more likely to be immigrants and injured as a result of the disaster. The prevalence rates of health problems for both MHS-users and non-MHS-users are listed in table 2 and 3. MHS-users were more likely to present psychological problems before the disaster compared to non MHS users.

**Table 1:** Characteristics of the study population (MHS users) and non-MHS users

	MHS (n = 339)	Non-MHS (n = 1197)
% Females	51.0	48.0
% Public insurance	86.1	70.4***
% Relocated	33.6	11.7***
% Immigrant	42.6	28.0***
% Injured	22.7	9.9***
% Single	12.1	11.4
Mean age (SD)	39.8 (13.3)	42.8** (15.2)

** p < .01, *** p < .001

**Table 2:** Prevalence rates of self-reported health problems 2–3 weeks and 18 months post-disaster

Self-reported health problems	MHS				Non-MHS			
		
	2–3 weeks post-disaster (T1)		18 months post-disaster (T2)		2–3 weeks post-disaster (T1)		18 months post-disaster (T2)	
		
	N	%	N	%	N	%	N	%
Depression (SCL-90-R)	128	47.2^2^	88	37.0^2^	182	17.0^2^	70	8.4^12^
Anxiety (SCL-90-R)	123	44.6^2^	86	35.7^12^	200	18.4^2^	58	6.9^12^
Hostility (SCL-90-R)	126	45.5^2^	93	37.8^2^	185	16.9^2^	60	7.1^12^
Somatisation (SCL-90-R)	95	34.2^2^	79	32.9^2^	129	12.0^2^	65	7.7^12^
Intrusions and avoidance reactions (IES)	250	90.6^2^	159	58.8^1^	729	67.3^2^	265	32.4^12^
Social functioning (RAND-36)	213	71.2^2^	120	47.2^12^	467	41.4^2^	168	19.2^12^

1. Statistically significant differences compared to the previous period within groups (X^2^; p < .01)

2. Statistically significant differences between groups within periods (X^2^; p < .01)

**Table 3:** Prevalence rates of physician diagnosed health problems 16 months pre-disaster and 18 months post-disaster

Physician diagnosed health problems	MHS				Non-MHS			
	16 months pre-disaster (P0)		18 months post-disaster (P1)		16 months pre-disaster (P0)		18 months post-disaster (P1)	
		
	N	%	N	%	N	%	N	%

Psychological problems	95	31.4^2^	272	84.0^12^	215	21.3^2^	546	50.3^12^
Chronic diseases	160	52.8	177	54.6	464	45.9	540	49.8^1^
Musculoskeletal problems	145	47.9	185	57.1^12^	416	41.1	483	44.5^12^
Gastrointestinal problems	82	27.1	105	32.4^2^	226	22.4	251	23.1^2^
Respiratory problems	87	28.7	101	31.2^2^	280	27.7	244	22.5^12^

1. Statistically significant differences compared to the previous period within groups (X^2^; p < .01)

2. Statistically significant differences between groups within periods (X^2^; p < .01)

### Predictors for post-disaster MHS use

To investigate factors associated with post-disaster MHS use socio-demographic and disaster variables were entered into the first regression model. Younger age, forced relocation, immigrant status, public insurance, unemployment, and being injured as a result of the disaster were significantly associated with MHS-utilization within 18 months post-disaster (table 4). Forced relocation and public insurance were also associated with MHS-use in a later period.

**Table 4:** Predictors for MHS use; adjusted Odds Ratios and 95% confidence intervals

Demographic and disaster-related variables	MHS use 0–18 months post-disaster (P1) n = 239		MHS use 18–36 months post-disaster (P2) N = 100		MHS use 0–36 months post-disaster N = 339	
	
	OR^1^	95% CI	OR^1^	95% CI	OR^1^	95% CI
Females	0.95	0.69–1.30	1.18	0.75–1.86	1.02	0.78–1.35
Age (in decades)	0.98	0.97–.99**	0.99	0.97–1.00	.98	0.97–0.99***
Relocation	2.42	1.70–3.47***	2.51	1.54–4.09***	2.98	2.16–4.13***
Immigrant	1.55	1.12–2.14**	1.33	0.83–2.13	1.57	1.18–2.09**
Single	0.80	0.47–1.35	1.33	0.69–2.56	0.95	0.61–1.48
Public insurance	1.49	1.00–2.22*	2.85	1.34–6.05**	1.86	1.30–2.67**
Unemployed	2.60	1.79–3.78**	1.95	0.96–3.99	2.38	1.75–3.95**
Injured	2.60	1.79–3.78***	1.29	0.71–2.35	2.49	1.75–3.55***

^1^adjusted for the other demographic- and disaster-related variables (sex, age, relocation, immigrant status, marital status, public insurance, unemployment, injuries); * p <.05, ** p < .01, *** p < .001

The results of the second regression model showed that a high score on the SCL-90-R subscales, RAND-36 social functioning subscale and IES were all significantly associated with MHS use in the subsequent period (see table 5). Furthermore, pre- and post-disaster musculoskeletal problems predicted MHS use respectively within and following 18 months post-disaster. Pre-disaster chronic diseases predicted MHS use within 18 months post-disaster and pre- and post-disaster physician diagnosed psychological problems were found to predict MHS use 18 months following the disaster (table 6).

**Table 5:** Predictors for MHS utilization; adjusted Odds Ratios and 95% confidence intervals

Self-reported health problems	MHS use 0–18 months post-disaster (P1)	
	
2–3 weeks post-disaster (T1)	OR^1^	95% CI
	
Social functioning problems (RAND-36)	2.14	1.53–2.99***
Anxiety (SCL-90-R)	2.53	1.78–3.59***
Depression (SCL-90-R)	3.05	2.14–4.33***
Somatisation (SCL-90-R)	2.47	1.70–3.59***
Hostility (SCL-90-R)	3.14	2.22–4.44***
Intrusions and avoidance reactions (IES)	4.04	2.37–6.91***
	
	MHS use 18–36 months post-disaster (P2)	
	
18 months post-disaster (T2)	OR^1^	95% CI
	
Social functioning problems (RAND-36)	2.97	1.74–5.07***
Anxiety (SCL-90-R)	2.17	1.12–4.22*
Depression (SCL-90-R)	2.08	1.07–4.07*
Somatisation (SCL-90-R)	2.34	1.20–4.56*
Hostility (SCL-90-R)	3.64	1.93–6.87***
Intrusions and avoidance reactions (IES)	2.67	1.39–5.14**

^1^adjusted for demographic- and disaster related variables (sex, age, relocation, immigrant, marital status, public insurance, unemployment, injuries); * p <.05, ** p < .01, *** p < .001

**Table 6:** Predictors for MHS utilization; adjusted Odds Ratios and 95% confidence intervals

Physician diagnosed health problems	MHS use 0–18 months post-disaster (P1)		MHS use 18–36 months post-disaster (P2)	
	
	OR^1^	95% CI	OR^1^	95% CI
16-0 months pre-disaster (P0)				

Psychological problems	1.33	0.92–1.91	1.75	1.07–2.86*
Chronic diseases	1.81	1.29–2.52**	1.20	0.75–1.93
Musculoskeletal problems	1.42	1.02–1.96*	1.25	0.78–1.99
Gastrointestinal problems	1.40	0.97–2.01	0.84	0.48–1.48
Respiratory problems	1.08	0.76–1.53	1.17	0.71–1.93

0–18 months post-disaster (P1)				

Psychological problems	na	Na	1.80	1.06–3.05*
Chronic diseases	na	Na	0.95	0.59–1.54
Musculoskeletal problems	na	Na	2.16	1.33–3.50**
Gastrointestinal problems	na	Na	1.25	0.76–2.07
Respiratory problems	na	Na	1.06	0.63–1.78

^1^adjusted for demographic- and disaster related variables (sex, age, relocation, immigrant, marital status, public insurance, unemployment, injuries); na: not applicable; * p <.05, ** p < .01, *** p < .001

In the third regression model, disaster intrusions and avoidance reactions and symptoms of hostility were significant independent predictors for MHS utilization 0–18 months following the disaster after adjustment for all other variables (table 7). Chronic diseases remained a significant predictor for MHS utilization within 18 months post-disaster. Although not statistically significant in table 6, ORs above 1.7 were observed for relocation, social functioning problems, public insurance and physician diagnosed musculoskeletal problems in P2 (ORs = 1.95, 1.79, 3.03 and 1.89 respectively), which might suggest that these factors are predictors.

**Table 7:** Multivariate logistic regression results of independent predictors for MHS use at P1 (0–18 months post-disaster) and P2 (18–36 months post-disaster)

Independent variables	MHS use		MHS use	
	
	P1		P2	
	
	OR^1^	95% CI	OR^1^	95% CI
Female	0.87	0.60–1.25	1.09	0.55–2.16
Age (in decades)	0.98	0.96–0.99**	0.98	0.96–1.00
Relocation	1.40	0.90–2.17	1.95	0.95–4.01
Immigrant	0.87	0.58–1.31	0.89	0.41–1.92
Single	0.75	0.40–1.40	0.50	0.14–1.71
Public insurance	1.05	0.66–1.67	3.03	0.75–5.52
Unemployed	1.23	0.61–2.51	1.40	0.40–4.87
Injured	1.80	1.13–2.86*	1.02	0.39–2.66
Social functioning problems (RAND-36)	1.61	1.05–2.44*	1.79	0.86–3.73
Anxiety (SCL90)	0.98	0.56–1.70	0.86	0.28–2.68
Depression (SCL90)	1.24	0.71–2.17	0.77	0.27–2.17
Somatisation (SCL90)	1.21	0.72–2.01	0.81	0.25–2.60
Hostility (SCL90)	2.04	1.28–3.25**	3.77	1.51–9.40**
Intrusions and avoidance reactions (IES)	2.86	1.48–5.53**	2.29	1.04–5.07*
Psychological problems (GP)	0.99	0.66–1.50	0.96	0.46–2.03
Chronic diseases (GP)	1.82	1.25–2.65**	0.99	0.50–1.99
Musculoskeletal problems (GP)	1.11	0.77–1.62	1.89	0.95–3.75
Gastrointestinal problems (GP)	1.20	0.79–1.82	1.04	0.50–2.17
Respiratory problems (GP)	0.80	0.53–1.21	0.80	0.37–1.71

^1^adjusted for demographic- and disaster related variables, self-reported health problems and physician diagnosed health problems

* p <.05, ** p < .01, *** p < .001

## Discussion

This study examined factors associated with post-disaster mental health service utilization in survivors of the Enschede fireworks explosion in The Netherlands. Our results provided evidence that demographic- and disaster related variables, self-reported symptoms and physician diagnosed health problems predicted MHS utilization after the disaster. Younger age, unemployment, immigrant status, low SES, forced relocation and personal loss/injuries as a result of the disaster were among the demographic- and disaster related variables predisposing for post-disaster MHS-utilization. Survivors who reported higher levels of emotional problems and problems on social functioning directly after the disaster were more likely to seek post-disaster MHS utilization. Regarding physician diagnosed health problems, pre-disaster psychological and musculoskeletal problems predicted post-disaster MHS use. When al variables were taken into account, disaster intrusions and avoidance reactions, symptoms of hostility and chronic diseases prior to the disaster were found to be the most important factors to predict post-disaster MHS utilization.

Our findings regarding the influence of demographic variables and disaster related variables on service utilization are to a large extent in line with what is found in earlier studies [1,9,14,27-31]. However, in our study we found an opposite effect for immigrant status (more likely to use MHS) in comparison to other studies [10]. Our finding that immigrants had a higher chance of using MHS was also found in other studies after this disaster where evidence was found that affected immigrants reported more psychological problems (before the disaster) and use MHS more often than native survivors [32,33]. A plausible explanation for the higher rates of MHS utilization among this group is that their higher rates of pre-disaster psychological health problems may be indirectly related to increased post-disaster MHS use. Possibly immigrants also displayed higher MHS use prior to the disaster. However, in our study pre-disaster MHS data were not available so we could not test this. Another explanation for the higher MHS utilization among immigrants in our sample could be found in the strategy of the MHS-unit which acted pro-actively with regard to minority groups. However, our definition of immigrants (first and second generation) is different from studies who use ethnicity and therefore can not be compared with [9,34-36].

Furthermore, we found in our study is that sex did not predispose for MHS use although former studies showed a positive relation for females [9,37]. Also, Dutch women use mental health services more often than their counterparts [38]. Another study among survivors of the same disaster found no differences in post-disaster psychological problems between men and women in general practice [39]. The absence of sex differences in help seeking behaviour may therefore be characteristic for this disaster.

We found that disaster intrusions and avoidance reactions and symptoms of hostility were important factors to predict post-disaster MHS utilization. The symptoms can be seen as the main clusters (re-experiencing, avoidance, arousal) of PTSD (according to the DSM-IV-criteria) which is found to be positively related to MHS use [10,11].

It is acknowledged that survivors presenting pre-disaster psychological problems are more at risk for psychological and physical health problems after the disaster [1,14,40,41]. Never reported before is our finding that survivors presenting physical health problems before the disaster are more likely to make use of MHS, even after controlling for confounders (table 6). Pre-disaster chronic diseases remained also an important independent predictor for post-disaster MHS-utilization after controlling for other variables (table 6). Our finding that somatic symptoms predict mental health seeking can be explained by the understanding that physical health problems are positively related to psychopathology and disability, and as a result of that, also to the need for mental health treatment. Another explanation for higher MHS utilization among individuals presenting chronic diseases is that they might be more likely to visit their general practitioner who diagnoses mental health problems and initiate treatment [11]. This finding implies that patients with chronic diseases are vulnerable during a disaster and therefore may be in need for mental support afterwards.

Several limitations should be addressed. The sample included a limited number of people as information from only one MHS unit was used. Persons visiting other health care providers (private psychologists and psychiatrists, inpatient institutions) were not included. This indicates that our results may be typical for the population who sought help in this unit. Although the study-population did not differ on demographic and disaster-related variables from MHS-patients who did not participate, it is possible that a selection has occurred, limiting the generalizability of the results. However, a study investigating selective participation in the health surveys of affected residents found that even though there was selective participation in the surveys, this did not affect the results [42]. Also our MHS data did not differentiate between the type of contact given (visits for preventive reasons, treatment) and treatment-time. A drawback of the study is that we did not measure pre-disaster mental health care utilization as the registration system was not running before the disaster. It is acknowledged that former treatment predicts post-disaster treatment [43]. The registration system in the MHS did not attain information on diagnoses (and therefore the diagnoses of PTSD could not be made), our finding underscores the importance of diagnosing in mental health services and in general practice (also for comparison purposes). Our 'non-MHS-group' was defined as disaster survivors who did not visit the specific MHS unit. However, it is possible they received post-disaster treatment of trauma in the private circuit. Further examination by self-reports 18 months post-disaster showed that around 5.6% of the disaster survivors in this group contacted a private psychologist/psychiatrist in the past 12 months for their disaster-related health problems.

The strength of our study is that we had a unique opportunity to combine survey data with medical records from general practitioners, allowing the collection of both subjective and objective information respectively. Besides, actual pre-disaster information on health status was available from the medical records. Having these pre-disaster data is rather unique in disaster research as most studies lack these data or are measured retrospectively which is more prone to recall bias. By using electronic records (EMRs of GPs and MHS) we excluded the possibility of respondents' recall bias and patients were not burdened in the data collections, which is an important issue after a catastrophic event. The present study fills a gap in disaster literature as most studies rely on self-reports only while in the present study both self-reported data and data from electronic medical records (containing pre-disaster data) were combined. Another strength of our study is that we studied a MHS-unit which was specially implemented for disaster-related treatment and all patients registered in this unit suffered disaster-related problems. GPs and health care professionals in the city were motivated to refer patients with disaster-related problems to this service.

## Conclusion

This study shows that demographic and disaster-related factors and psychological and somatic health problems predict post-disaster MHS utilization. However, the most important factors that predispose for post-disaster MHS utilization are disaster intrusions and avoidance reactions and symptoms of hostility (which can be identified as symptoms of PTSD) and pre-disaster chronic diseases. This implies that survivors experiencing chronic diseases before the incident are especially vulnerable during a disaster and therefore may be in need for mental support afterwards.

The implementation of electronic registration systems in health services is important for future disaster-studies as it provide important information on pre-disaster health status. Putting information from both self-reports and electronic registration systems into a multivariate framework allows us to correct for potential confounders which adds to the existing literature on predictors for MHS utilization. An important direction for future research is the investigation of PTSD and the medical conditions.

## Competing interests

The author(s) declare that they have no competing interests.

## Authors' contributions

All authors read and approved the final manuscript. DJO drafted the design, analysed and interpreted the data and drafted the manuscript. PGV and LG supervised the data collection from the surveys and participated in preparation of the manuscript. MM made substantial contributions to data analysis, interpretation of data and preparation of the manuscript. AJED participated in the design and assisted in the preparation of the manuscript. CJY supervised the data collection from the electronic medical records and assisted in the preparation of the manuscript.

## Pre-publication history

The pre-publication history for this paper can be accessed here:


